# Fighting the invisible enemy: providing support and structure to radiology resident during the COVID-19 pandemic

**DOI:** 10.1590/0100-3984.2020.0067

**Published:** 2020

**Authors:** Regina Lucia Elia Gomes, Luisa Leitão de Faria, Hernane Ajuz Holzmann, Natalia Kimie de Faria Fujiwara, Sabrina de Mello Ando, Marcio Valente Yamada Sawamura, Claudia da Costa Leite, Giovanni Guido Cerri

**Affiliations:** 1 Instituto de Radiologia do Hospital das Clínicas da Faculdade de Medicina da Universidade de São Paulo (InRad/HC-FMUSP), São Paulo, SP, Brazil.

**Keywords:** Pandemics, Radiology, Internship and residency, Pandemias, Radiologia, Internato e residência

## Abstract

The objective of this article is to share the strategy we used in order to restructure the radiology and diagnostic imaging department of a referral institution during the coronavirus disease 2019 pandemic, on the basis of the current recommendations. It is essential to integrate the work of supervisors, preceptors, and residents, maintaining communication and sharing decisions, with mutual support, as well as to determine the best strategy to be adopted in this scenario of uncertainty and constant change, while also ensuring adequate emotional support for all parties.

## Abstract (pt)

O objetivo deste artigo é compartilhar a estratégia de como estruturamos o departamento de radiologia e diagnóstico por imagem de uma instituição de referência na pandemia da COVID-19, baseados nas recomendações vigentes. É fundamental a integração entre supervisores, preceptores e residentes, sempre mantendo comunicação e compartilhamento das decisões, com apoio mútuo, decidindo a melhor estratégia a ser seguida neste cenário de incertezas e de mudanças contínuas, garantindo ainda suporte emocional adequado a todos.

## INTRODUCTION

Throughout the world, radiology residency programs have adapted to the coronavirus disease 2019 (COVID-19) pandemic by working together with their supervisors and preceptors to develop a strategy to combat the pandemic while ensuring the safety and well-being of resident physicians, who constitute a healthy workforce with increasing demands and are always trying to continue their training^(1)^. On the basis of the experiences of other countries, we managed to prepare for this new reality in Brazil, seeking to provide guidance for everyone involved.

Even before the beginning of the COVID-19 pandemic in Brazil, measures were taken to provide support and structure for resident physicians, in accordance with the recommendations of the Brazilian National Ministry of Health, the Brazilian National Ministry of Education and Culture (MEC), the São Paulo state government, the Clinical Board of the Hospital das Clínicas da Faculdade de Medicina da Universidade de São Paulo, the Comissão Nacional de Residência Médica (CNRM, Brazilian National Medical Residency Commission), and the Comissão de Ensino de Aperfeiçoamento e Residência (CEAR, Commission for the Advancement of Teaching and Residency Programs) of the Colégio Brasileiro de Radiologia e Diagnóstico por Imagem (CBR, Brazilian College of Radiology and Diagnostic Imaging). Preceptors (chief residents) play a fundamental leadership role in residency programs, bridging the gap between program supervisors and residents, helping them achieve their goals and develop their skills, as well as dealing with the other peculiarities of each program; in this period of uncertainty, preceptors became even more important in the preparation and maintenance of responses to the demands of COVID-19^(2)^.

As mandated by the government of the state of São Paulo, in decree 64862 of March 13, 2020^(3)^, vacations were canceled for attending physicians and residents during the pandemic, to be rescheduled for a later date. The objective of the decree was to keep the workforce at full strength during the height of the crisis.

The program supervisors and preceptors, working jointly, developed a strategic plan to confront the pandemic, the first planning session having been conducted on March 15, 2020. The objective of disseminating the plan was to be able to share the experience even in that phase at which the incidence of COVID-19-related illness and death had not peaked in Brazil, when the disease was still spreading across various Brazilian cities and states.

Circular no. 1/2020, issued by the CNRM and MEC on March 19 and ratified by the CEAR-CBR on March 25, guided our strategy^(4,5)^. To reorganize the resident schedules for a period of three months during the pandemic, our strategic planning took into consideration the following aspects (Table 1): location, comorbidities, volunteers to work on the front lines, rotations, training in COVID-19 imaging, and online activity tools. The objective was to minimize the effects of the pandemic on instruction in radiology while promoting the care of patients with COVID-19, focusing on an emotional support plan during the crisis.

**Table 1:** Strategic planning for three months and the respective measures taken.

Strategic planning for three months	Measure
Location	Division into high- and low-risk areas
Comorbidities	Diabetes mellitus, hypertension, asthma, obesity, immunosuppression, and other factors taken into consideration
Frontline clinical work	Voluntary adherence, with replacement of teams every five weeks
Rotation	Performed separately in the high- and low-risk areas
Training in COVID-19 imaging	Provision of materials and testing, at the beginning and end of March, prior to the peak of the pandemic in Brazil
Online activity tools	Classes, report releases, seminars, case discussions, etc.
Emotional support	Increased frequency of virtual mentorship and creation of the COMVC-19 support group

## LOCATION

The various institutes of Hospital das Clínicas were divided into areas of higher and lower risk, depending on the presence or absence of patients with COVID-19, respectively. With that in mind, we began to organize the division of resident physicians into two groups-one assigned to work in the high-risk area and one assigned to work in the low-risk area-each allowing monthly rotations of residents only within the group. In addition, unlike our previous open-door policy regarding the radiology reporting rooms, we restricted access to the reporting rooms at all institutes, providing telephone extensions to clinicians and surgeons, in case they had questions, thus maintaining the recommended social distancing. Radiologists thus remained in their important role as consultants, always willing to help colleagues from other specialties in the analysis and interpretation of imaging findings, sharing their knowledge for the benefit of patients.

## COMORBIDITIES

Residents with a personal history of risk factors for COVID-19, as defined by the World Health Organization and the Brazilian National Ministry of Health^(6)^, including hypertension, diabetes mellitus, asthma, obesity, heart disease, lung diseases, and other comorbidities, were directly assigned to the low-risk area.

## VOLUNTEERS

Of the 115 radiology residents and fellows, 65 (56.5%) were assigned to work in the high-risk areas. Of the 71 residents, 12 (17%) volunteered to work in the clinical care of patients with COVID-19. Before starting their activities in clinical care, all of the volunteers were trained by the team of the Residency Program in Internal Medicine, the objective being to standardize all procedures adopted by the institution and to update the radiology residents regarding best practices. The 65 residents and fellows were in the rear guard in the emergency room or in other high-risk areas, such as the ultrasound and computed tomography sectors.

## ROTATIONS

Rotations were scheduled within the high-risk and low-risk areas, so that each group of residents remained in their respective area, in order to avoid contamination and protect the residents with risk factors for COVID-19 or other comorbidities. A new schedule was created for a three-month period, establishing one-month rotations within the high-risk and low-risk areas, again with no crossover between the two. Over the three-month period, the rotations were performed, the groups at greatest risk being those in the emergency department, the reporting room, and the computed tomography scan console (for incidental COVID-19 patients), as well as those performing bedside ultrasound examinations in COVID-19 patients, in wards and intensive care units, with access to examinations from the various specialties, the emphasis being on urgent cases and findings related to COVID-19. The low-risk rotations were performed at other institutes, the emphasis being on the imaging areas of pediatrics, oncology, neurology, orthopedics, cardiology, and pulmonology . The entire team of attending physicians made themselves available for in-person instruction.

Radiologists and radiology residents did not perform their activities remotely (from home), as was done at other institutions in Brazil and abroad. Our decision to require these professionals to work at the hospital took into account the fact that radiologists are working on the front lines in the fight against COVID-19, like other physicians, thus standardizing the participation of physicians from various specialties, all of whom performed their activities in person, regardless of whether or not they were capable of working remotely^(7)^. The supervisors and preceptors instructed the residents to divide themselves into two shifts, to reduce the number of people who circulated in the reporting rooms and in other common areas of the radiology department, such as workstations rooms, the clinical staff room, and the library, to which the workstations were moved, in order to avoid overcrowding. The first shift started at 7:00 a.m. and ended at 2:00 p.m. (including a lunch break), and the second shift started at 2:00 p.m. and ended at 8:00 p.m., so that only half of the usual residents were present at any given time. In the middle of each monthly rotation, the groups switched shifts. Residents who so desired could also remotely accompany the activities carried out during the other shifts.

## TRAINING IN COVID-19 IMAGING

All 115 radiology residents and fellows were trained previously (in March) to identify the main imaging aspects of COVID-19, especially thoracic findings, although also less common findings, such as those seen on abdominal and neurological imaging examinations. The training, which included findings on X-ray, ultrasound, and computed tomography of the chest, was carried out by selecting recent articles on the topic and activities offered online, as well as classes given by our faculty in Imagine, the first official course on the radiology calendar, which is innovative because it is completely virtual in 2020^(8)^. Subsequently, the preceptors applied a test to assess performance. Through the use of institutional videos, the residents and fellows were also trained in the donning and removal of personal protective equipment.

## ONLINE ACTIVITY TOOLS

The online tools WhatsApp and Workplace are already enshrined in the routine of the radiology residency program. During the pandemic, we started a new, specific WhatsApp group-COVID-InRad-so that the supervisors and preceptors could closely monitor the volunteer resident physicians during their performance on the front lines, providing consistent support. During the pandemic, there were rotations of the volunteers in order to reduce emotional exhaustion and to include all radiological activities. However, the volunteers who left the front lines of clinical care remained in the group, contributing to the support of new volunteers by sharing their acquired experience.

To reduce crowding, teaching activities were carried out remotely, albeit still within the hospital, with Google Meet, a tool previously adopted by the institution. All of those teaching activities were carried out during the shifts of the residents. Google Meet facilitates the release of examinations by sharing the computer screen of the picture archiving and communication system, allowing comments on and discussion of the most relevant findings, as well as making it possible to view corrections to the previous report and the final report subsequently issued. It also makes it possible to record the activity performed, in order to make it available to the volunteers who are working in clinical care, so that they can review the report later. In addition, a number of videoconferences have been held for each of the specialties in the rotation, as have general meetings, case presentations, and seminars, in order to reduce the impact of the pandemic. Large national and international radiology organizations have also provided free online teaching materials, which are aimed at consolidating the theoretical training^(9,10)^. In relation to the pre-pandemic period, there was an increase in the use of online educational activities, which can certainly be used later for the resumption of activities, enabling greater integration among all parties, despite the distances between the institutes.

## EMOTIONAL SUPPORT

The Mentorship Project of the Department of Radiology of the Hospital das Clínicas and the support group of the Hospital das Clínicas Institute of Psychiatry employee mental health care program, known as COMVC-19, have provided essential emotional support to the residents, as have the supervisors and preceptors. Social detachment and isolation can have negative psychological impacts of the residents, especially given the fact that many are not from the city of São Paulo and have therefore been apart from their families during this pandemic. There are a number of stress factors that can lead to burnout, fatigue, and depression among residents, including the reduction in the number of outpatient examinations scheduled, the impact of the pandemic on teaching activities, the avoidance of hospitals on the part of the public, which can result in a smaller volume of cases for the learning of the specialty, and the chance of being reassigned to a high-risk area of the hospital.

Residency program supervisors should have private meetings with residents to determine whether there are any personal, social, or health problems that might put their families at risk. For example, a resident or a family member of a resident may be immunocompromised. That would inform decisions on how to allocate residents^(9,11,12)^.

### The Mentorship Project

The Department of Radiology of the Hospital das Clínicas Mentorship Project has existed for almost four years and aims to help achieve success in career and personal life through monthly meetings between groups of residents and their respective mentors. In a proactive measure aimed at improving emotional support, the project was intensified during the pandemic, the meetings being held on a weekly rather than monthly basis and being conducted online rather than in person.

## COMVC-19

The psychiatric and psychological support group of the Hospital das Clínicas Institute of Psychiatry, established more than 25 years ago, extended its activities to all hospital employees, as well as to university students and resident physicians, working intensely to provide guidance and to detect cases of burnout, fatigue, or depression as early as possible. The employee mental health care program (COMVC-19) involves activities to promote health, as well as to prevent and treat reactions to stress and mental disorders. The program also has a training component for residents and health care professionals focused on mental health care, aiming to expand activities to monitor mental health, risk factors, and protective factors among employees, thus promoting the well-being and the safety of all parties, including non-medical staff^(13,14)^.
